# Effect of Rare Earth Element Ce on Nanoscale (Ti, Nb) C Precipitates and Mechanical Properties of High-Strength Low-Alloy Weathering Steel

**DOI:** 10.3390/ma18133033

**Published:** 2025-06-26

**Authors:** Yunlong Wang, Rui Zhu, Hairui Ma, Guohua Ding, Limeng Liang, Weiwei Sun, Yongxia Wang

**Affiliations:** 1 School of Mechanical and Vehicular Engineering, Bengbu University, Bengbu 233030, China; 2 Anhui Engineering Research Center of Additive Manufacturing, Bengbu 233030, China

**Keywords:** weathering steel, rare earth elements, precipitation strengthening, microstructure evolution, mechanical properties

## Abstract

This study investigates the influence of rare earth element Ce addition on the nanoscale precipitation, microstructure, and mechanical properties of Ti-containing secondary phases in high-strength low-alloy weathering steel. Mechanical property testing and microstructural characterization were performed on experimental samples subjected to rolling-aging treatment. The results demonstrate that the addition of Ce promotes coarsening of nanoscale precipitates, thereby diminishing their precipitation strengthening effect. At a 0.11% Ce content, an increase in inclusions was observed, leading to crack formation during hot deformation. However, Ce addition also refines inclusion size and modifies inclusion types, contributing to steel purification. Through austenite recrystallization zone rolling combined with an isothermal process, a high-strength ferritic weathering steel with nanoscale precipitates was fabricated, exhibiting a yield strength of 635 MPa, tensile strength of 750 MPa, and elongation of 21.2%. Precipitation strengthening plays a critical role in enhancing the room-temperature strength of ferritic steel.

## 1. Introduction

The new generation of high-strength low-alloy (HSLA) weathering steels is developed through microalloying design and advanced thermomechanical controlled processing (TMCP) based on conventional low-alloy steels, exhibiting superior performance as engineering structural materials [1,2]. Currently, these steels have been extensively applied in various engineering fields including railway transportation, bridge construction, and marine applications. With the ongoing development trends of railway vehicles toward higher speed, heavier load capacity and lower manufacturing cost, there is an increasing demand for steels with enhanced strength, improved corrosion resistance and better cost-effectiveness in railway carriage and shipping container manufacturing [3]. Improving steel strength to reduce vehicle weight has become a crucial approach to meet these technical requirements. Consequently, replacing conventional low-strength steels with high-strength atmospheric corrosion-resistant steels has emerged as an inevitable choice in industrial applications. Presently, weathering steels with 450 MPa yield strength have been widely implemented in 70 t and 80 t-grade railway freight cars [4]. However, the application of higher strength grade (≥550 MPa) atmospheric corrosion-resistant steels remains relatively limited in China’s railway freight vehicle industry, particularly for heavy-haul transportation applications. This limitation primarily stems from challenges in achieving optimal combinations of mechanical properties and corrosion resistance in higher strength grades.

Precipitation strengthening and fine grain strengthening are effective means to improve the strength and toughness of steel [5,6,7,8]. Nb, V and Ti are commonly used micro-alloying elements, while Ti is rich in China, and the cost of Ti alloying is the lowest, which has obvious advantages for the production of low-cost steel plates. Ti forms carbonitride with C and N, which is dispersed in the matrix to play the role of precipitation strengthening and can also refine the grains. The precipitation strengthening effect is directly affected by the size and volume fraction of the precipitated phase, especially the size of precipitated phase. The smaller the size of the precipitated phase, the better the precipitation effect. When the size of the precipitate is tens of nanometres, the strengthening effect is weak, and the service temperature increases; therefore, the ‘ pinning’ effect is small, and only the size of the carbonitride precipitated phase is less than 10 nm, which can play a significant role in precipitation strengthening.

The research on nano-scale precipitates is one of the hot issues in the current strengthening methods. In 2004, JFE Steel [5] developed a Ti-Mo ferrite-based steel with a tensile strength of 800 MPa. This steel increases the precipitation strengthening to about 300 MPa by precipitating unit nano-scale carbides on the ferrite matrix. The steel has high strength and good formability. Cai et al. [6] pointed out that Ti-Nb nano-precipitates can improve the yield strength and tensile strength of the martensitic matrix without affecting the elongation of the steel. In the field of refractory materials [7], the precipitation strengthening effect of Ti-Mo nano-precipitates can compensate for the loss of matrix strength of refractory steel in a high temperature environment. The addition of Mo element can improve the solid solubility of Ti, Nb (C, N) in austenite, so that a large amount of Nb remains in the solid solution and is dispersed in the low temperature transformation, resulting in a higher precipitation strengthening effect. It can also increase the nucleation position of carbonitrides, so that the formed carbides are more dispersed and finer. Cheng et al. [8] successfully developed ultrafine-grained ferritic steel with excellent comprehensive mechanical properties (average grain size of 1.1 µm) by using nano-scale precipitation combined with large deformation low temperature rolling technology.

Current technological advancements have achieved nanoscale control of carbide precipitates in steel, though further size reduction presents diminishing returns in cost-effectiveness. Metallurgical systems inevitably contain coarse polygonal TiN precipitates alongside Al_2_O_3_-CaO-MnS composite inclusions formed during the addition of deoxidizers like Al and Ca. As demonstrated by Xue et al. [9], heterogeneous nucleation phenomena occur when multiple oxide inclusions coexist in molten steel, with Ti-Nb precipitates preferentially forming on Al_2_O_3_ substrates through epitaxial growth, ultimately developing into oversized composite inclusions. This mechanism aligns with findings by Wang et al. [10], who revealed MnS’s propensity to nucleate on Al_2_O_3_ particles, resulting in characteristic Al_2_O_3_-MnS hybrid structures.

Crucially, inclusion size exerts exponential effects on material performance: Experimental evidence indicates a two-order-of-magnitude reduction in fatigue life with a mere doubling of inclusion dimensions [11]. Systematic investigations [12,13] confirm that minimizing inclusion size in bearing steels significantly enhances both impact toughness and fatigue resistance, underscoring the critical importance of inclusion refinement in high-performance steel design.

Large-scale inclusions frequently serve as initiation sites for pitting corrosion in weathering steels, as extensively documented in studies [14,15,16]. The strategic incorporation of rare earth elements (REEs) in alloyed steels demonstrates significant metallurgical benefits: REEs preferentially react with sulfur and oxygen, effectively reducing deleterious Mn/Al-based inclusions while generating finely dispersed rare earth composite inclusions [17]. This microstructural modification not only optimizes inclusion morphology through uniform dispersion but also concurrently enhances mechanical strength and corrosion resistance [17,18,19]. Specifically, cerium addition demonstrates dual functionality—passivating active corrosion sites through cathode/anode reaction suppression [20] and modifying inclusion chemistry.

Notably, corrosion initiation mechanisms vary with elemental composition. Kim et al. [21] identified (Mn,Cr,Fe)-O-S dissolution as the primary pitting trigger in duplex stainless steels, contrasting with rare earth-modified systems. Park et al. [19] revealed that Ce alloying enhances pitting resistance through two synergistic mechanisms: promoting Cr-rich zone formation while suppressing Cr-depleted regions, and redirecting corrosion propagation from stable Ce oxides to α-phase grain boundaries. This phenomenon is corroborated by observations in rare earth-modified 25% Cr duplex stainless steel, where pitting initiates at the interface between (REM,Cr,Mn)-O-S inclusions and the metallic matrix [18].

Despite these advancements, critical knowledge gaps persist. The fundamental mechanisms governing rare earth alloying remain incompletely understood, particularly regarding its influence on nano-scale precipitation behavior in Ti-Nb-Mo-containing high-strength low-alloy weathering steels [22]. Furthermore, the absence of standardized technical protocols for quality control in rare earth-modified steel production continues to hinder industrial implementation. These unresolved challenges underscore the need for systematic investigations into rare earth metallurgy to fully exploit its potential in advanced steel design.

Building upon China’s unparalleled rare earth reserves, this work demonstrates an economically viable pathway for steel performance enhancement through cerium modification. Our systematic investigation of Ce’s triple functionality—precipitation refinement, matrix optimization, and property enhancement—in Ti-containing weathering steels provides both scientific insights and practical guidelines for developing next-generation steel materials with Chinese technological signatures. The findings establish fundamental principles for rare earth utilization while addressing critical quality control challenges in industrial-scale production.

## 2. Material and Methods

### 2.1. Test Materials and Methods

**Figure 1:**
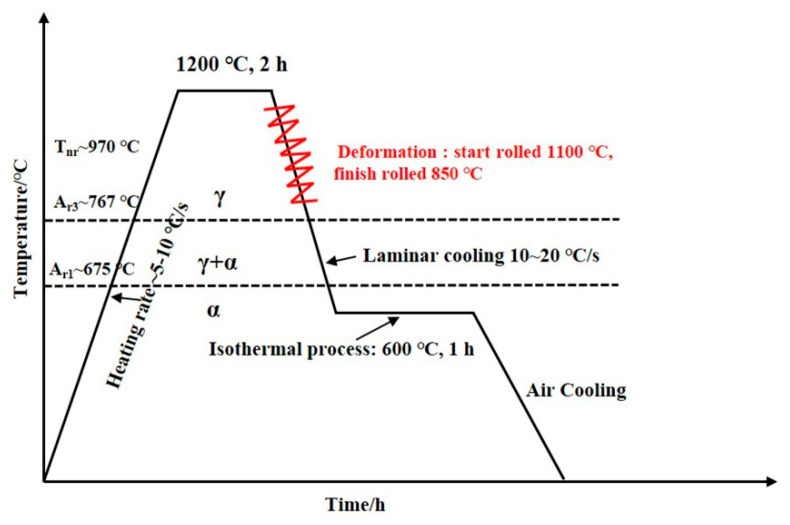
Experimental steel rolling process roadmap.

Table 1 details the chemical composition of the experimental steels. The three kinds of experimental steels were forged with a length of 1.2 m and a cross-sectional size of 80 × 80 mm. To facilitate the hot rolling experiment, they were evenly divided into three sections using a sawing machine. Specimens underwent reheating at 1200 °C for 2 h homogenization prior to thermomechanical processing. Multistage hot rolling was conducted on a Φ350 two-high mill (University of Science and Technology Beijing) through controlled reduction sequences, i.e., 80 → 60 → 50 → 40 → 30 → 20 → 15 → 10 mm (Figure 1), with deformation strategically distributed across both austenite recrystallization and non-recrystallization regimes.

Post-rolling cooling employed laminar flow technology to rapidly quench plate surfaces to 600 °C. Processed plates subsequently underwent 1 h isothermal holding in a pre-calibrated furnace followed by ambient air cooling. Real-time temperature monitoring was implemented using infrared pyrometry to ensure thermal consistency throughout the experiment.

**Figure 2:**
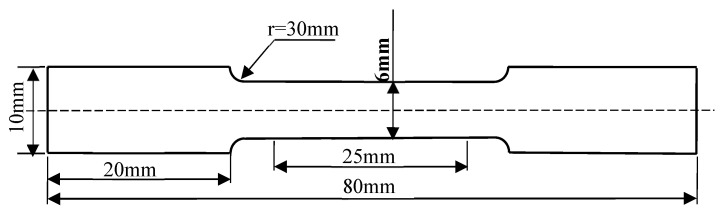
Schematic diagram of tensile test dimensions.

Tensile characterization of the hot-rolled steel plates was conducted using a CMT 5605 universal testing system (Institute of Engineering Technology, University of Science and Technology Beijing) following ASTM-compliant protocols (GB/T 228.1-2010 [23]). The mechanical property test specimens selected in this paper are rod-shaped tensile specimens. The sampling position is in the middle part of the rolled plate. The specimen dimensions are shown in Figure 2.

Cylindrical tensile specimens, machined along the rolling direction, underwent quasi-static loading at a controlled strain rate of 1 mm/min. To ensure statistical robustness, triplicate tests were performed for each material condition, with ultimate tensile strength (UTS) and elongation values calculated as arithmetic means of three independent measurements.

### 2.2. Microstructure Characterization Methods and Parameters

After the rolling experiment, three samples with a size of 10 mm × 10 mm × 5 mm were cut on each steel plate. The microstructure of the samples was observed and analyzed by an optical microscope (OM, Zeiss AX10, Jena, Germany) and laser confocal scanning microscope (CLSM, Keyence VK-X250, Osaka, Japan). The OM microstructure samples were ground on silicon carbide paper. After polishing, the samples were ultrasonically cleaned with anhydrous ethanol, dried, and eroded with 4% nitric acid alcohol solution for 5~10 s. Then, the samples were quickly washed with deionized water, ultrasonically cleaned in anhydrous ethanol, dried, and placed in a dry dish for use.

The microstructure and inclusion morphology of the test steel were observed and analyzed by using the scanning electron microscope of Quanta FEI 450 of the Engineering Technology Research Institute of Beijing University of Science and Technology (Beijing, China). The composition and distribution of inclusions were analyzed by Oxford EDS energy dispersive X-ray spectroscopy equipped on a scanning electron microscope (SEM).

To elucidate the role of cerium (Ce) in modulating nanoparticle precipitation within the experimental steel, site-specific nanoscale specimens were fabricated using a dual-beam focused ion beam scanning electron microscope (FIB-SEM; Thermo Fisher Scientific Helios G4 CX, Waltham, MA, USA). These atomically sharp needle-shaped samples (<100 nm tip radius) were subsequently analyzed via laser-assisted three-dimensional atom probe tomography (3D-APT) to resolve elemental distributions with sub-nanometer spatial resolution. This correlative microscopy approach enables direct linkage between Ce-containing precipitates identified in SEM and their compositional profiles at the atomic scale.

## 3. Results

### 3.1. Microstructure Characteristics

**Figure 3:**
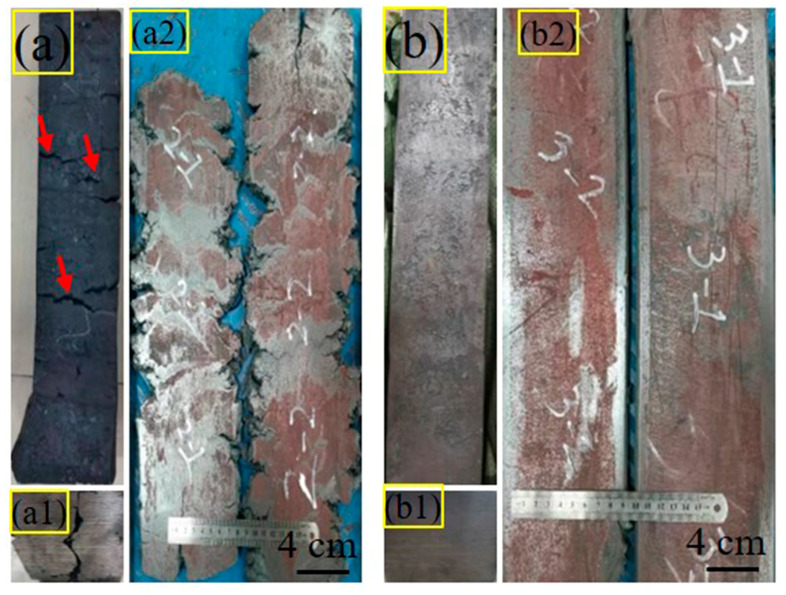
Forgings and rolled plates: (**a**) 0.11 CE-TI steel forgings; (**a1**) 0.11 CE-TI forging section; (**a2**) 0.11CE-TI rolled plate (**b**) 0.012CE-TI forging; (**b1**) 0.012 CE-TI forging section; (**b2**) 0.012 CE-TI rolled plate.

As illustrated in Figure 3, three distinct test steel plates with varying Ce-Ti compositions demonstrate significantly different surface characteristics following hot rolling processes. The 0CE-TI and 0.012CE-TI specimens maintained smooth surfaces throughout rolling operations, while the 0.11CE-TI steel plate exhibited severe cracking during processing. This phenomenon can be attributed to the intentional addition of 0.11% Ce during the smelting process, which induced the formation of numerous rare earth oxide inclusions within the steel matrix. These inclusions significantly compromised the material’s plastic deformation capacity, manifesting initially as forging defects that subsequently propagated conspicuously during subsequent rolling operations. The exacerbated crack propagation observed during rolling stages underscores the critical influence of Ce content on the mechanical integrity of steel alloys during thermomechanical processing [18,24].

**Figure 4:**
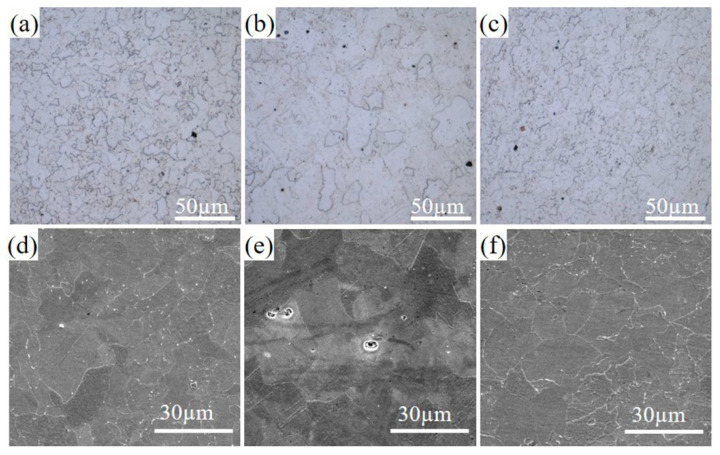
OM and SEM of hot rolled microstructure of three steels: (**a**,**d**) 0CE-TI; (**b**,**e**) 0.11CE-TI; (**c**,**f**) 0.012CE-TI.

Figure 4 presents the microstructural characteristics of the three experimental steel grades following hot rolling. All specimens exhibited a ferrite-dominated matrix, with microstructural analysis revealing a combination of limited quantities of fine deformed ferrite and abundant polygonal ferrite structures. This microstructural evolution arises from thermomechanical processing parameters: The hot rolling temperature within the austenite recrystallization zone-initiated deformation-activated recrystallization of austenite during rolling, with a final rolling temperature of approximately 850 °C. At this elevated temperature, recrystallized grain boundaries underwent coalescence, leading to coarsening of recrystallized austenite grains [5,25]. Subsequent laminar cooling to 600 °C preserved these recrystallized austenite grains while facilitating ferrite nucleation and growth within this thermal regime.

Distinct metallurgical behavior occurred in non-recrystallized grains subjected to deformation within the austenite–ferrite two-phase region. These grains demonstrated deformation-induced phase transformation (DIFT), characterized by the preferential formation of ultrafine ferrite grains at grain boundaries and high dislocation density regions. The concurrent phase transformation during deformation in the two-phase region suppressed ferrite grain growth during cooling, resulting in stabilized fine ferrite structures. This dual-pathway mechanism—combining recrystallization-driven grain coarsening and DIFT-enabled refinement—ultimately produced the observed composite microstructure dominated by polygonal ferrite with localized regions of refined grains.

### 3.2. Morphology of Inclusions and Precipitates

**Figure 5:**
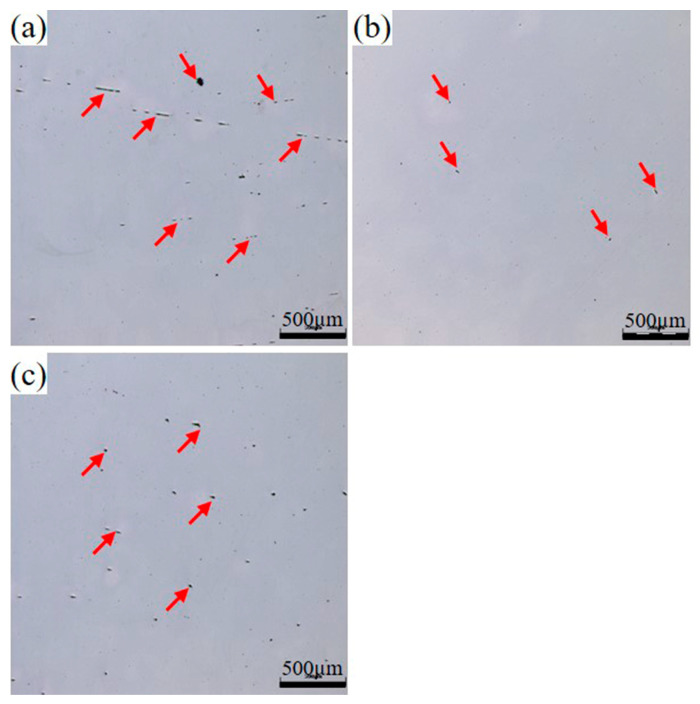
Macroscopic inclusion distribution diagram of experimental steel: (**a**) 0Ce-Ti; (**b**) 0.012Ce-Ti; (**c**) 0.11Ce-Ti.

Figure 5 is the low-magnification OM inclusion morphology of the three experimental steels. The size and density distribution of inclusions in 0.012 CE-TI samples containing Ti-Ce were lower than those in 0CE-TI and 0.11 CE-TI samples. The size of inclusions in 0CE-TI was the largest. Combined with the EDS composition analysis below, it is not difficult to see that the inclusions of 0.11CE-TI and 0.012CE-TI are mainly Al_2_O_3_ and TiN, and the inclusions of 0CE-TI are mainly Al_2_O_3_, TiN, MnS and (Ca, Mg) O introduced in the smelting process. The inclusions of the three steels mostly exist in the form of composite inclusions.

**Figure 6:**
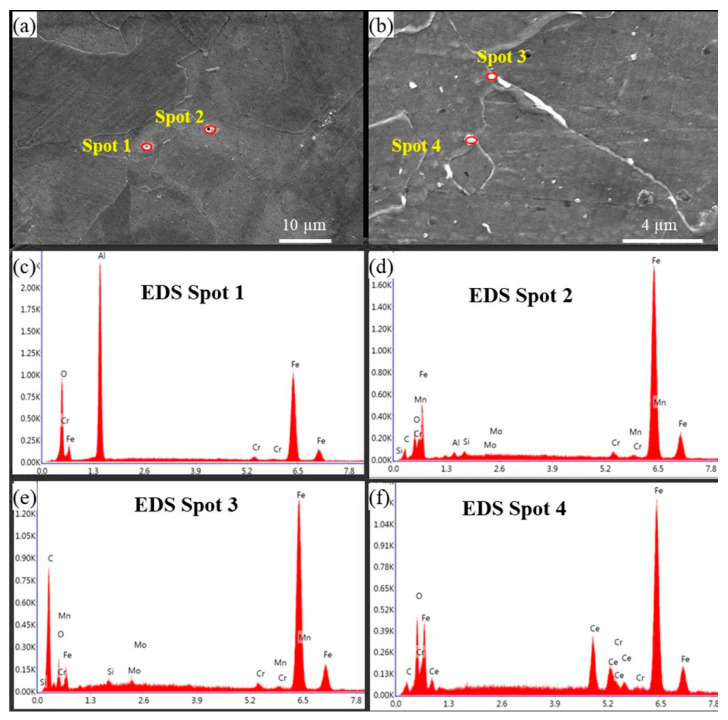
EDS point scanning of inclusions in samples: (**a**) 0Ce-Ti; (**b**) 0.11Ce-Ti, (**c**) EDS result at spot 1, (**d**) EDS result at spot 2, (**e**) EDS result at spot 3, (**f**) EDS result at spot 4.

Under the electron microscope, it was observed that the white particles were dispersed on the sample structure, and the particles were locally amplified and analyzed by the EDS energy spectrum. The results are shown in Figure 6. The white particles in the 0CE-TI sample were composed of alumina inclusions and carbides and oxides in the steel. The white particles in the 0.11CE-TI sample were mostly Ce-containing fine inclusions and composite inclusions formed by fine strip carbides and oxides at the grain boundaries.

### 3.3. Mechanical Properties

**Figure 7:**
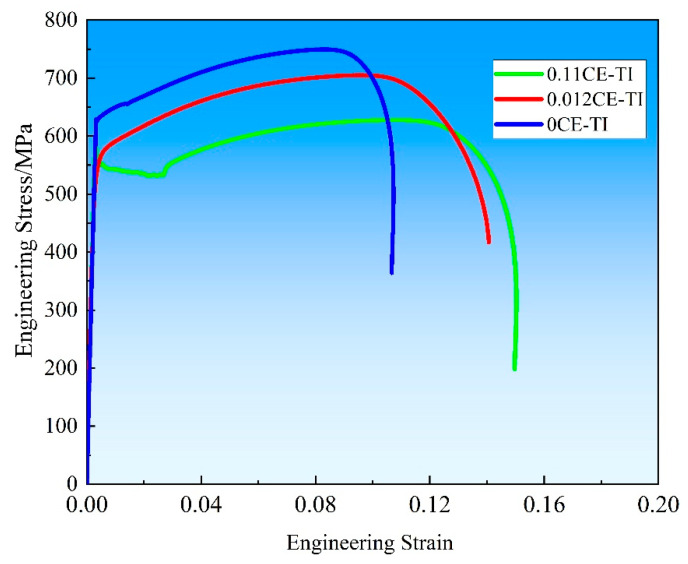
Tensile curves of three experimental steels at room temperature.

Figure 7 is the room temperature tensile curves of three kinds of experimental steels obtained by the rolling process. For the 0Ce-Ti sample, UST = 750 MPa, YS= 635 MPa, and ε = 21.2%. For the 0.012Ce-Ti sample, UST = 705 MPa, YS = 562 MPa, and ε = 22.6%. For the 0.11Ce-Ti sample, UST = 630 MPa, YS = 550 MPa, and ε = 24.6%. By comparing the mechanical properties of different steels at room temperature under the same rolling conditions, it was found that the addition of rare earth element Ce has a harmful effect on the mechanical properties. 0CE-TI experimental steel has the best tensile properties under the same rolling process. According to previous studies [26,27,28], there are many reasons affecting the tensile properties of steel at room temperature, such as microstructure, grain size, precipitate state and texture state.

**Figure 8:**
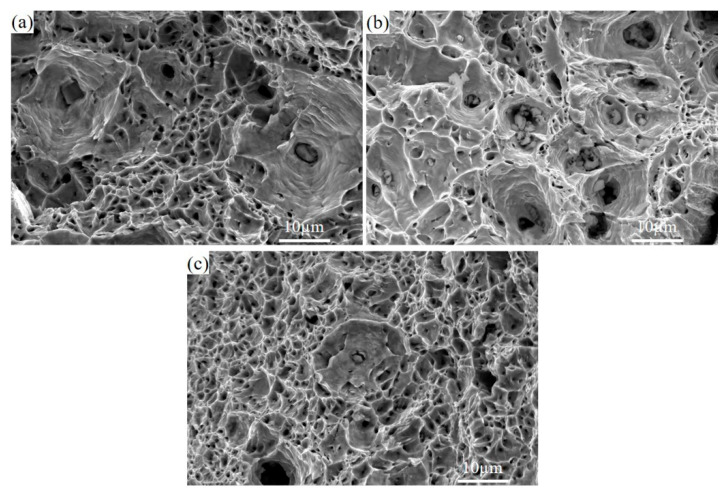
Tensile fracture morphology characteristics: (**a**) 0CE-TI-A sample fracture; (**b**) 0.11Ce-Ti specimen fracture; (**c**) fracture of 0.012Ce-Ti specimen.

The specimens demonstrate obvious necking during tension. The local enlarged diagram shows that a large number of dimples are distributed on the fracture surface, and there are inclusions in the dimples, as shown in Figure 8. During the tensile process, inclusions will act as stress concentration to promote the formation and expansion of dimples and accelerate the fracture of steel samples. As far as the current smelting level is concerned, it is not easy to completely remove inclusions in steel. Therefore, the number and size of inclusions should be reasonably controlled in the smelting process, which will be conducive to improving the comprehensive mechanical properties of materials.

## 4. Discussion

### 4.1. Modification and Purification of Rare Earth Elements

**Figure 9:**
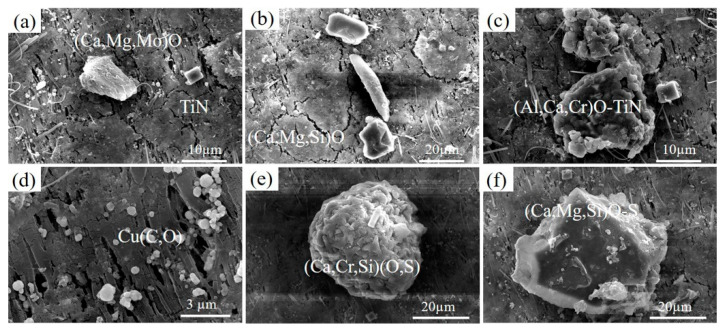
Three-dimensional morphology of 0CE-TI inclusions: (**a**) Ca, Mg and Mo complex oxides; (**b**) Ca, Mg and Si complex oxides; (**c**) composite inclusions of Al, Ca, Cr oxides and TiN; (**d**) Cu(C,O) inclusions; (**e**) composite inclusions of Ca, Cr, Si oxides and sulfides; (**f**) composite inclusions of Ca, Mg, Si oxides and sulfides.

**Figure 10:**
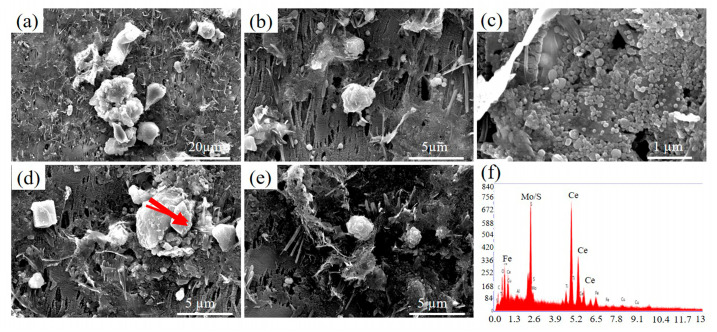
Three-dimensional morphology of 0.012 CE-Ti inclusions: (**a**) composite inclusions containing Ce and TiN; (**b**) composite oxides containing Ce; (**c**) morphology of a large number of fine CE-containing inclusions; (**d**,**e**) morphology of Ce inclusions, TiN composite inclusions and TiN inclusions; (**f**) morphology of fine Ce inclusions.

The three-dimensional morphology of inclusions in steel was obtained by electrolysis, as shown in Figure 9 and Figure 10. Through comparative observation, it is found that the addition of the Ce element obviously reduces the size and type of inclusions, and the effect of purifying molten steel is evident. There are large-sized Ca-Mg-S-O composite oxides in 0CE-TI steel. The 0.012CE-TI steel containing Ce is mostly fine Ti-Ce inclusions. The size of the inclusions has an effect on the corrosion. The chemical concentration difference between the large-sized inclusions and the matrix is large, which is more likely to cause the dissolution of the surrounding matrix or itself to form pitting corrosion [29]. 

**Figure 11:**
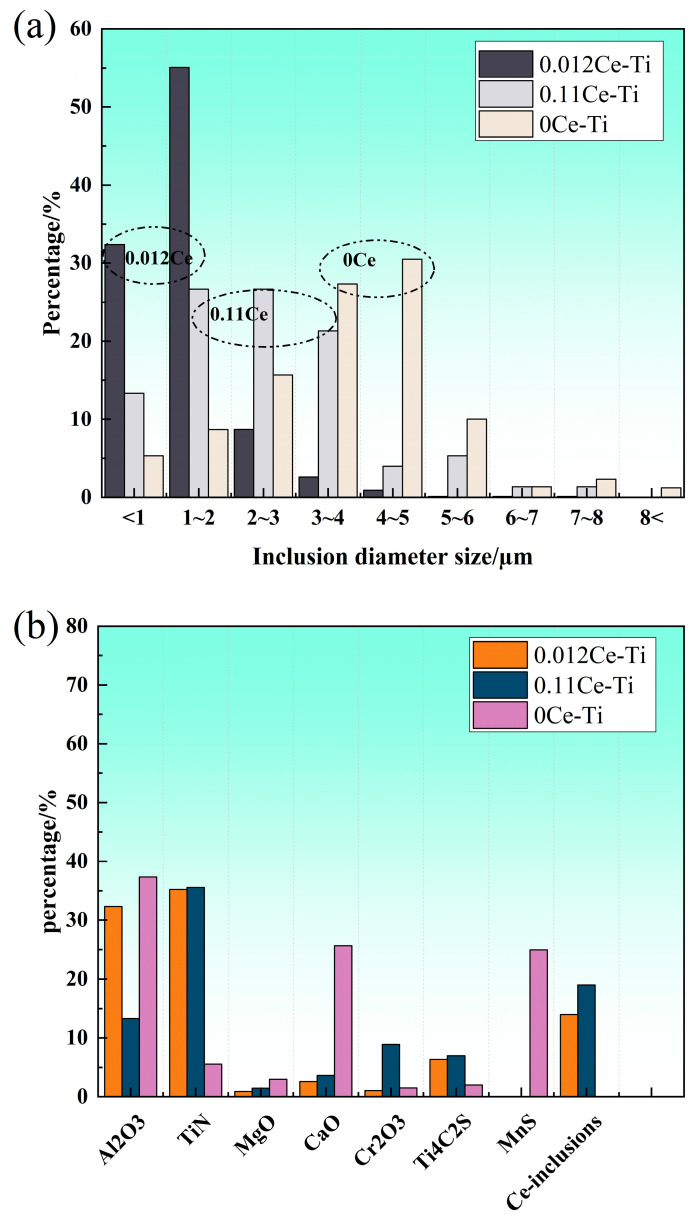
Inclusion size distribution and proportion diagram: (**a**) inclusion size distribution diagram; (**b**) proportion of inclusion species.

Figure 11 shows that the size and density distribution of inclusions in the 0.012CE-TI sample containing Ti-Ce are lower than those in the 0CE-TI sample. The size of inclusions in 0CE-TI is the largest. The inclusions of 0.11CE-TI and 0.012CE-TI are mainly composed of Al_2_O_3_ and TiN, and the inclusions of 0CE-TI are mainly composed of Al_2_O_3_, TiN, MnS and (Ca, Mg) O introduced in the smelting process. The inclusions of the three steels mostly exist in the form of composite inclusions.

The inclusions in 0CE-TI are irregular in shape. From the observation of the morphology and element distribution of the inclusions, it is not difficult to see that the content of O and Al in the middle of the inclusions is higher, while the content of S and Mn at the edge of the inclusions is higher. The main inclusions in the 0CE-TI matrix are Al_2_O_3_-MnS-TiN composite inclusions. In 0.11CE-TI containing Ti, i.e., Al_2_O_3_-TiN, TiN is mainly distributed at the edge of the inclusion. Due to the addition of Ti, a large number of S elements are attracted by Ti to form titanium sulfide inclusions, and no MnS inclusions are found in nearly 20 SEM fields of view. After the coordinated addition of Ti-Ce, the size and number of inclusions in 0.012CE-TI are significantly reduced, and the inclusions are nearly spherical Ce_2_O_2_S inclusions or demonstrate regular multi-deformation morphology. In contrast to the other two steels, TiN inclusions can completely coat CeAlO_3_-Ce_2_O_2_S inclusions.

### 4.2. Atomic-Scale Characterization of Ce-Induced Precipitation Behavior via 3DAP Analysis

**Figure 12:**
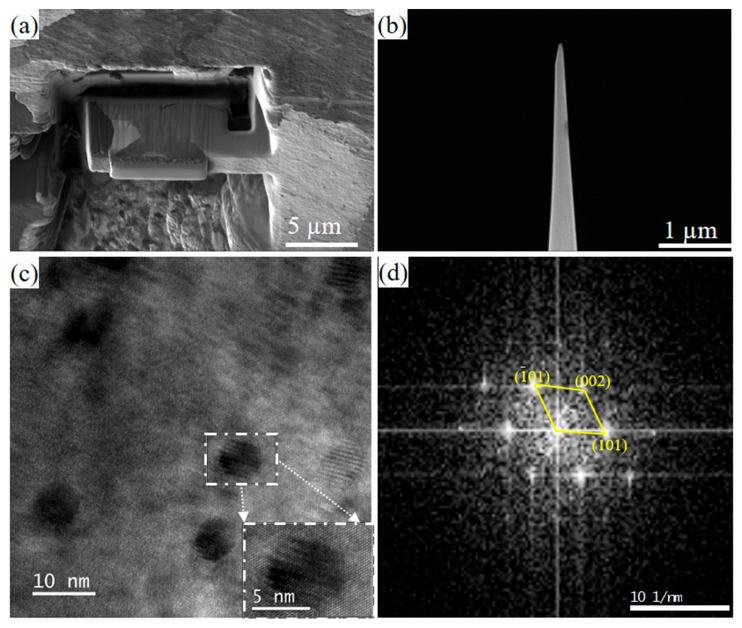
0CE-TI experimental steel 3DAP sample preparation diagram and TEM characterization of nano-precipitates in 0.012CE-TI steel: (**a**) cutting position; (**b**) needle tip sample. (**c**) HRTEM image of 0.012CE-TI (**d**) corresponding to the diffraction spot at the white rectangle below.

In order to study the effect of alloying elements on nanoprecipitation, 3DAP experiments were used to observe the morphology and analyze the composition of nanoprecipitation. Figure 12 is the sample preparation position of the 0CE-TI sample and the tip sample cut by FIB. Figure 12c shows the HRTEM image of the sample 0.012CE-TI. The white rectangular box in the picture is the outline of the nano-precipitate. Fourier transform was performed on the selected area in the white rectangular box to obtain the diffraction spots of the nanoprecipitates, as shown in Figure 12d. The results show that the nanoprecipitates are mainly TiC.

**Figure 13:**
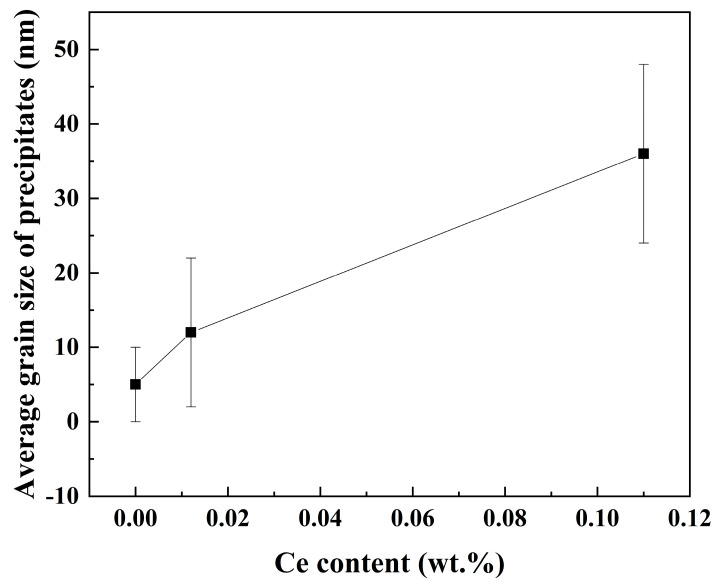
Variation in average size of nano-precipitates with Ce content.

The average grain size is shown in Figure 13. It is not difficult to see that with the increase in rare earth element Ce content, the size of nano-precipitated phase particles gradually increases. The size of nano-precipitates in the three samples is about 6~45 nm. Especially when the Ce content is 0.11%, the precipitated phase of the rolled sample in the amorphous region can reach more than 40 nm. Among them, the average size of nano-precipitates in the 0Ce-Ti sample is 5 nm, 12 nm for 0.012Ce-Ti and 36 nm for 0.11Ce-Ti. The relative atomic radius of the Ce atom is 0.1877 nm, and that of the iron atom is 0.1210 nm. The maximum lattice gap of the face-oriented cubic structure of Austenitic surfaces is 0.0352 nm [18,30].

Therefore, through the analysis of atomic size and crystal structure, the solid solubility of Ce in steel is very weak. Therefore, free Ce atoms are mainly concentrated at the grain boundaries or phase interfaces, occupying the diffusion channels of solute atoms by taking advantage of defects, hindering the diffusion of carbon atoms and other solutes, weakening the carbon emission capacity of austenite, inhibiting the formation of oxide and sulfide inclusions, and promoting the nucleation and maturation of nano-precipitated phases.

**Figure 14:**
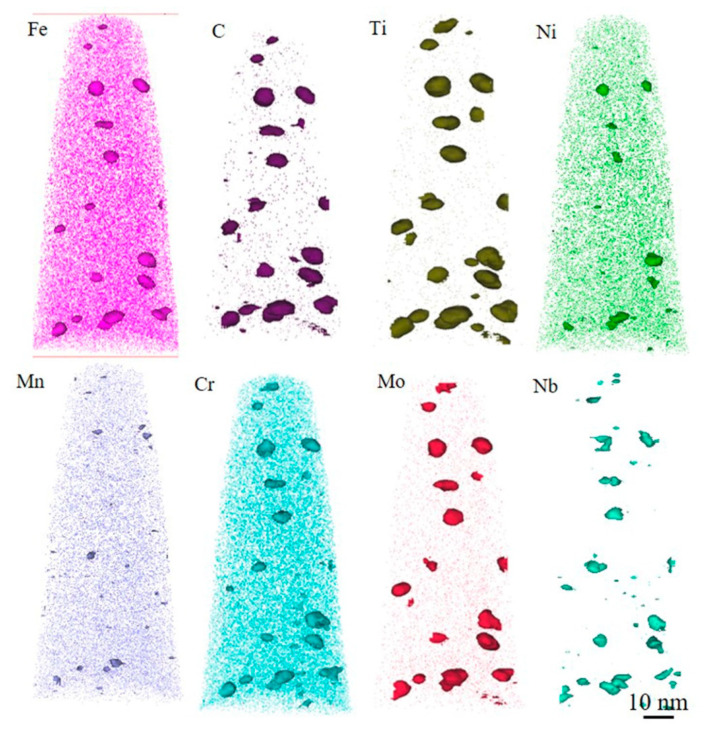
The element distribution diagram of 3DAP tip of 0CE-TI experimental steel.

**Figure 15:**
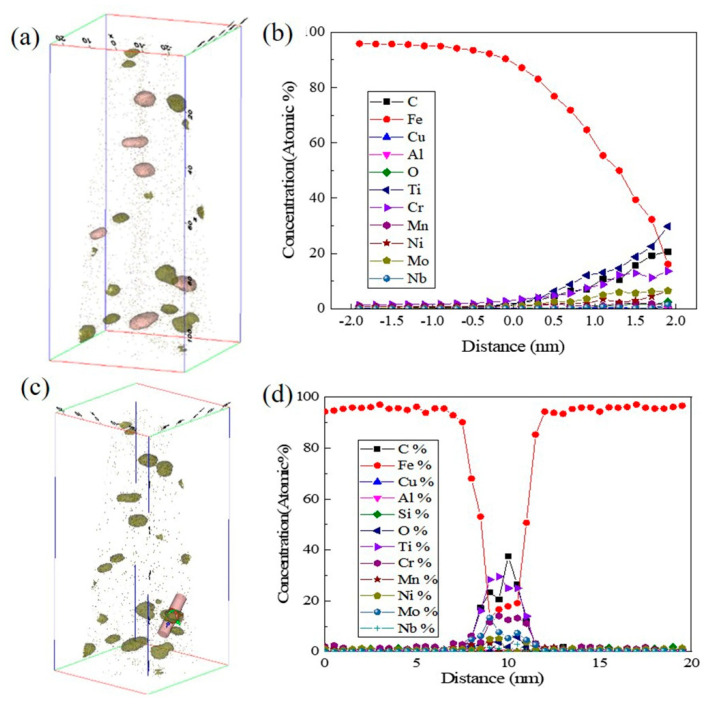
0CE-TI experimental steel nano-precipitates’ three-dimensional morphology and element distribution: (**a**) precipitate morphology; (**b**) the average element content change curve of the pink marker precipitate; (**c**) single precipitate composition analysis cylindrical mark; (**d**) element distribution in cylindrical region.

Figure 14 shows the element distribution of the 0CE-TI sample. It is found that the precipitate is mainly composed of C, Ti, and contains alloy elements such as Cr, Mo, Nb and Fe. From the morphology observation, the precipitates are mostly irregular near ellipse, with a size of about 1–10 nm. It is found that the chemical composition distribution of the same precipitate is not uniform. Compared with the size of the element enrichment zone at the same precipitate position, it was observed that the content of Fe, Mo, Ni and Cr in the core of the precipitate was higher and the size was smaller, but the edge of the precipitate was dominated by Ti and C elements. The average element content from the surface to the core of the nanoprecipitates is shown in Figure 15b. It can be seen that the content of Ti and C gradually increases from the matrix to the edge of the precipitate, and the concentration of Ti tends to be stable at about 1/4, and the concentration of Cr, Mo, Ni and other alloy elements in the center increases. The composition analysis of a single nano-precipitated particle was carried out. The results are shown in Figure 15d. The concentration of Fe and Cr elements in the core of the precipitate was significantly higher than that in the edge. The concentration peak of Mo element appeared at 1/4, and the enrichment phenomenon also appeared in the core.

**Figure 16:**
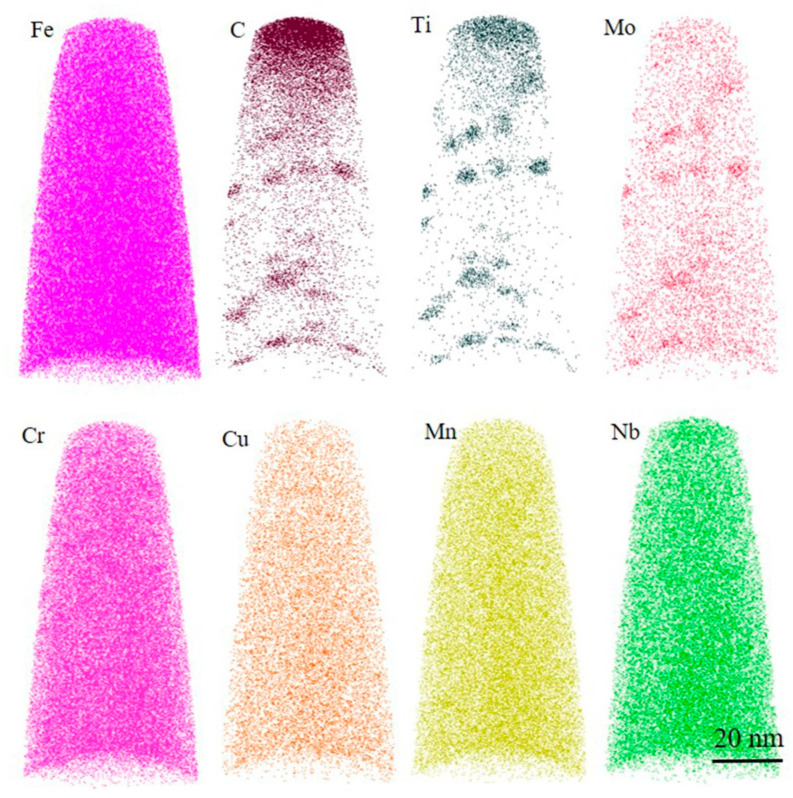
Element distribution diagram of 3DAP tip of 0.012CE-TI experimental steel.

**Figure 17:**
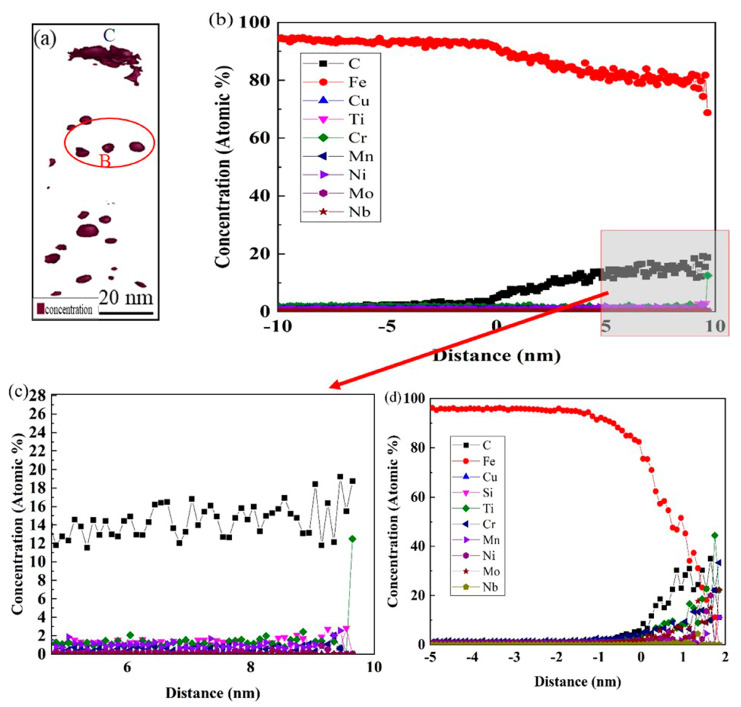
Three-dimensional morphology of nano-precipitated phase in 0.012CE-TI experimental steel and element distribution: (**a**) morphology of precipitated phase; (**b**,**c**) the average content of precipitates at position B; (**d**) the content of large-size precipitates at position C.

Figure 16 shows the element distribution of the 0.012 CE-TI sample. The test results did not detect the characteristic peak of Ce element, which was related to its low content and low solid solubility in steel. It was found that the main components of the precipitate were Fe, C, Ti and Mo. The chemical composition of the precipitate was different from that of 0CE-TI, and the proportion of Fe element increased significantly. The size of precipitates was significantly larger than that of 0CE-TI, and some large-sized precipitates were more than 20 nm. The separate composition analysis of large-size precipitates shows that the core of large-size precipitates is also enriched in alloying elements such as Cr, Mo and Ni, but the content of Ti element is the largest, as shown in Figure 17d. The average content analysis of small-sized nano-precipitates showed that the concentration of Cr element in the core of the precipitates increased significantly, and the proportion of Fe element also increased compared with 0CE-TI steel. Based on the results of 3DAP, it is found that Mo elements in 0CE-TI and 0.012CE-TI steels are enriched in the core of nano-precipitates, which promotes the dispersion of TiC in steel.

### 4.3. Effect of Precipitation Strengthening on Mechanical Properties

The yield strength of microalloyed steel is generally improved by solid solution strengthening, fine grain strengthening, precipitation strengthening and dislocation strengthening. The yield strength *σ_y_* can be expressed by the following formula [31,32]:

*σ_y_* = *σ_0_* + *σ_s_* + *σ_g_* + *σ_p_* + *σ_d_* (1)

where *σ*_0_ is the dislocation lattice resistance of pure iron; *σ*_0_ is closely related to the shear elastic modulus (G) of pure iron; *σ_g_* is the amount of fine grain strengthening; *σ_s_* is the amount of solid solution strengthening, which is related to the mass fraction of alloying elements in steel; *σ_p_* is the amount of precipitation strengthening; *σ_d_* is the dislocation strengthening amount.

Because the main alloy components of the three steels are similar, and the matrix structure is the same (ferrite), the contribution of solution strengthening and dislocation strengthening to the three steels is similar [33]. This is a very reasonable estimate. This paper focuses on the difference in the contribution of fine grain strengthening and precipitation strengthening to the strength of three experimental steels [31]. *σ_g_* is the amount of fine grain strengthening, which is related to the effective size of grains. The grain refinement strengthening can be estimated using the experimentally determined Hall–Petch equation [7,33]:

*σ_g_* = *k_y_* × *d*^−1/2^ (2)

where *k* is a constant and *k_y_* = 17.4 MPa mm^1/2^;

*d* is the average grain size, μm.

According to Formula (1), the effective *σ_p_* is *σ_y_*− (*σ*_0_ + *σ_s_* + *σ_g_* + *σ_d_*). The value of Formula (2) can be determined according to the average grain size of the upper section. The average grain size is introduced into the formula, and the contribution values of fine grain strengthening of 0CE-TI, 0.11CE-TI and 0.012CE-TI steel rolled in austenite recrystallization two-phase zone are 218 MPa, 175 MPa and 205 MPa, respectively. Compared with the previous tensile mechanical properties, it is not difficult to find that precipitation strengthening plays an important role in the mechanical strength of steel.

## 5. Conclusions

(1) High-strength ferritic weathering steel with nanoscale precipitates was successfully fabricated via austenite recrystallization zone rolling, achieving YS = 635 MPa, UTS = 750 MPa, and EL = 21.2%. Precipitation strengthening contributed ~300 MPa to yield strength, demonstrating its critical role in ferritic steel strengthening.

(2) The addition of rare earth Ce refined inclusion size and modified inclusion types (Al_2_O_3_-MnS → Ce_2_O_2_S-TiN), effectively purifying the steel matrix. For optimal performance, we recommend a Ce content of 0.012–0.03 wt.%—sufficient to achieve inclusion refinement while minimizing precipitate coarsening.

(3) Excessive Ce (0.11 wt.%) promoted undesirable effects: precipitate coarsening (growth to >40 nm), reducing precipitation strengthening efficiency by ~25%; formation of brittle Ce-Al-O inclusions, inducing cracking during hot deformation. Regarding trade-off requirements, industrial applications must balance corrosion resistance (favored by higher Ce) with mechanical properties (favored by lower Ce).

## Figures and Tables

**Table 1:** Actual chemical compositions of the experimental steels (wt.%).

Alloy	C	Si	Mn	P	S	Ti	Cr	Ni	Cu	Mo	Nb	Al	Ce
0CE-TI	0.050	0.26	0.70	0.004	0.0036	0.10	1.32	0.34	0.35	0.15	0.031	0.014	0
0.012CE-TI	0.045	0.25	0.66	0.004	0.0040	0.10	1.29	0.30	0.35	0.15	0.032	0.015	0.012
0.11CE-TI	0.051	0.26	0.70	0.004	0.0034	0.08	1.28	0.33	0.350	0.15	0.035	0.015	0.11

## Data Availability

The original contributions presented in the study are included in the article, further inquiries can be directed to the corresponding author.
